# Draft Genome Sequence of *Pseudomonas aeruginosa* ATCC 33988, a Bacterium Highly Adapted to Fuel-Polluted Environments

**DOI:** 10.1128/genomeA.01113-14

**Published:** 2014-11-06

**Authors:** Lisa M. Brown, Thusitha S. Gunasekera, Oscar N. Ruiz

**Affiliations:** a University of Dayton Research Institute, Dayton, Ohio, USA; b Air Force Research Laboratory, Aerospace Systems Directorate, Fuels and Energy Branch, Wright-Patterson AFB, Ohio, USA

## Abstract

*Pseudomonas aeruginosa* ATCC 33988 is highly adapted to grow in jet and diesel fuel, with a defined regulation of adaptive genes and metabolization of *n*-alkanes. The draft genome of strain ATCC 33988 is 6.4 Mb in size, with 5,975 coding sequences and 66.3% G+C content, and it is highly similar to that of the clinical strain *P. aeruginosa* PAO1.

## GENOME ANNOUNCEMENT

*Pseudomonas aeruginosa* ATCC 33988, also known as *P. aeruginosa* QMB 1592, was isolated from a fuel storage tank in Ponca City, OK. This strain efficiently metabolizes *n*-alkanes and grows extremely well in Jet-A fuel and F-76 diesel (1). Transcriptional profiling demonstrated that *P. aeruginosa* ATCC 33988 is highly adapted to the fuel environment and is able to regulate multiple metabolic pathways to enhance its resistance to fuel (2). The wealth of information on the regulation and metabolism of *P. aeruginosa* ATCC 33988 and its high capacity for hydrocarbon degradation compelled us to define its genetic makeup by sequencing its genome.

*P. aeruginosa* ATCC 33988 was sequenced on a Roche 454 GS Junior platform using a whole-genome shotgun (WGS) approach, resulting in 388,306 reads. The sequence reads were assembled with the Roche *de novo* Assembly software. The assembly reported 224 large (>500 bp) contigs, with an *N*_50_ of 68,712 bp. The longest contig was 235,965 bp. The draft genome sequence is 6,410,803 bases in length, with a G+C content of 66.3%, which is similar to the 66% G+C content of the *P. aeruginosa* PAO1 strain. Rapid genome annotation using the RAST annotation server (3) described 5,975 coding sequences (CDSs) and 60 structural RNAs, which consist of one 16S rRNA, one 23S rRNA, and 58 tRNAs. The coding sequences were classified into 564 subsystems, of which amino acids and derivatives (*n* = 716 CDSs), carbohydrates (*n* = 458), cofactors (*n* = 360), vitamins and pigments (*n* = 360), protein metabolism (*n* = 279), cofactors and membrane transport (*n* = 307), cell wall and capsule (*n* = 220), fatty acids, lipids, and isoprenoids (*n* = 212), stress response (*n* = 193), iron acquisition and metabolism (*n* = 146), virulence, disease, and defense (*n* = 142), metabolism of aromatic compounds (*n* = 125), and miscellaneous (*n* = 88) were the most abundant subsystems. These results support that this strain is well adapted to cope with biotic and abiotic stresses, including fuel contamination.

A comparison of *P. aeruginosa* ATCC 33988 with other *P. aeruginosa* strains within the RAST database identified *P. aeruginosa* 19BR as its closest neighbor, with a score of 501, followed by *P. aeruginosa* 213BR, with a score of 490. *P. aeruginosa* PAO1 (taxonomy ID 208964.1) was the twelfth closest neighbor, with a score of 343.

The *P. aeruginosa* ATCC 33988 transcriptome shows high similarity with the PAO1 strain, with hybridization to >5,460 probe sets contained within the PAO1 GeneChip Pae_G1a (2). A DNA sequence comparison of the genes related to the adaptation of *P. aeruginosa* ATCC 33988 to fuel, including alkane degradation (*alkB1* and *alkB2*), efflux pumps and porins (*oprF, oprG, mexC, mexD, oprJ, mexE, mexF*, and *oprN*), biofilm formation (*pelA, pelD, pelE*, and *algD*), and iron acquisition (*fur, pvcD, pvDE, pchF*, and *pfeR*) were at least 99% identical to those in PAO1. Further analyses showed *alkB1* and a*lkB2* have two synonymous single-nucleotide polymorphisms (SNPs) compared to the a*lkB1* and *alkB2* genes of the PAO1 strain.

### Nucleotide sequence accession number.

This whole-genome shotgun project has been deposited in DDBJ/EMBL/GenBank under the accession no. JPQQ00000000.
